# President Stille’s Opening Address

**Published:** 1871-06

**Authors:** 


PRESIDENT STILLE'S OPENING ADDRESS.

The President commenced his address by remarking that exactly a quarter of a century ago a convention assembled in New York City for the purpose of founding this Association.

When these events took place, California had but recently been brought within the boundaries of the United States. This State, which now draws travelers from every quarter of the globe to admire its sublime scenery, its marvelous vegetation, and its fabulous wealth, was known to few beyond its borders, and excited but little interest anywhere. Separated from the populous regions of the East by ranges of lofty mountains, by boundless prairies, and by arid deserts, it seemed destined to perpetual isolation, or to be approached only by a tedious and circuitous ocean voyage.

But twenty-five years ago! Yet to-day civilization sets by the Golden Gate in a populous and splendid city; industry and wealth teem over the land so lately trodden only by the savage or the indolent Mexican; the devious voyage and the dreary and dangerous journey of the slow caravan are replaced by the swift cars that bring the Atlantic and Pacific shores within a week of one another. The features of this young capital announce her culture of art; her literature proves that she sprang full-grown from the brain of civilization; our meeting here to-day proclaims that her physicians and her medical institutions are in harmony with those of the medical world beyond the mountains.

While this precocious development is, perhaps, less an example of growth from the seed than of transplantation of the natural product, it is, nevertheless, to be observed that the intellectual growth of California is paralleled by the extraordinary productions of its soil, whose flowers, and fruits, and forest trees excel in magnitude and exuberance those of the eastern shore of our continent. And we may fairly anticipate that the climate of this western land, which already has marked the inhabitants with a healthier hue, and subdued somewhat that adent and restless temperament which distinguishes Eastern Americans, will peculiarly fit them for the investigation and solution of the higher problems of medicine.

There is something so marvellous in our assembling here as almost to make one doubt the evidence of his senses. That one of the largest of the learned Associations of the country should hold its sessions where, a quarter of a century ago, the words science and art had never been pronounced appears miraculous. And yet, if we regard the history of our own profession, the feeling of astonishment will hardly be less, for we shall see that during the same period it has also advanced with scarcely shorter strides upon the pathway of improvement.

The President enlarged upon this subject and then continued:

Among the instruments devised for hastening the progress of popular and professional enlightenment, is this Association. It was founded by physicians who were painfully alive to the deficiencies of the schools, especially in comparison with those of Europe. Some who were most ardent in labor for establishing a higher grade of medical education, had resided abroad, and had felt humiliated in comparing their own attainments with those of their foreign contemporaries. Others who had not been subjected to this painful experience were, nevertheless, acquainted with our shortcomings, and were equally in earnest in arousing the medical prcfession and inducing the medical colleges to enlarge their curriculum, prolong their terms of study, and exact from candidates for the medical degree a larger amount of professional knowledge. There was no doubt of the need of improvement, nor any of the sincerity of those who advocated it. It is very certain, also, that the combined action of 1846, which created this body, only represented the culmination of partial movements towards the same object which had taken place during at least thirty years before. The schools and the profession were as one in conviction and desire. * * *

Why, then, is it that although the profession and schools are agreed in the essential principles involved in the problem of medical education, so little should be practically done to solve it? This question will, perhaps, be best answered by contrasting once more our own country with others in which a systematic and elaborate method is established. The first contrast which strikes us is that everywhere, except in the United States, medical, like all other education, is regulated by law; so that neither the number of schools, nor the duration of pupilage, nor the extent of the curriculum, nor the condition of graduation, can be arranged to suit the intellectual culture of any particular locality, nor the convenience of any particular faculty. Before the late Franco-German war the number of medical schools in France was only three, in Germany and Austria together not more than a dozen, and in Great Britain and Ireland about the same number. Each one of these geographical divisions contains a population as great as that of the United States, if not greater, and yet we number about five times as many medical colleges as do the largest Empires of Europe. Admitting that our vast territory justifies, in some degree, but only in a small degree this disparity (for distance has almost been annihilated by railroads), it cannot be doubted that we support at least three times, perhaps ten times, as many medical colleges as we require. And while this excess remains, it is in vain to expect that all, or even a majority, of the schools will agree in adopting an improved plan of education. The interests of the greater number of them forbid it imperatively. * * * * *

It is evident, therefore, that no progress can or will be made in the true direction unless one of two preliminary conditions is secured. Either some one institution must be endowed so as to be rendered independent of its rivals, or a number of the leading schools must agree together to adopt a curriculum in harmony with the present state of medicine, and with the system of instruction pursued in the principal schools of the world. Of two conditions there seems no prospect whatever that the first can be fulfilled. The execution of the second depends entirely on the goodwill of the colleges that are interested in the decision. No one can act alone; and every effort to induce several of them to enter into a compact which shall be of mutual obligations, and one not to be abrogated without the consent of all the contracting parties, or at least a large majority of them, has hitherto proved unavailing. What motives, if any, will determine the adoption of a different policy may be conjectured, but need not be suggested; yet it is safe to affirm that if the profession at large were to lend their support to those colleges and only those which determine to carry out essentially the recommendations of the conventions of medical teachers held at Cincinnati in 1867, and at Washington in 1870, we should soon enjoy the benefits of a system of education which would place the American medical profession upon a perfect equality with that of the most favored country.

It cannot be denied that the view which has now been presented of the condition and future requirements of medical education, demands a certain sort of abstraction. The seeker after truths must take a position from which his eye can observe, not only the past, nor the present, nor an imaginary future alone; he must embrace them all in a comprehensive view, and discern the law under which phenomena stand, or as consequences of other phenomena. In this manner he will be liable to recognize them collectively as links of a chain that stretches from the beginning to the end of time, and learn that however little it may flatter his pride as an inventor or discoverer, his highest as well as most useful office is to build upon the foundation of the past, and according to the plan which the wisdom of ages, which is the highest experience, has determined to be the best.

To the judicious mind, there is in this no humiliation, for it is done in obedience to a natural law which pervades and controls all progress, whether social, civil, literary, or scientific. The perfected creature of to-day may have passed through countless ages of progressive development, and, in like manner, the germs of scientific truth have by slow degrees unfolded, casting of errors as useless appendages to the higher organism. How vast the space between the experiment of Galvani and the science which now bears his name! How wide the distance between the juggleries of Mesmer and the science of nerve physiology! How great the gulf between the charlantry of Hahneman and the splendid edifice which is now growing out of a study of the natural history of disease! Nothing exists in vain. Even fraud teaches by warning, and the world has furnished examples of prevalent errors which concealed some germ of truth. Like the wheat buried with an Egyptian mummy, it springs to life when once it is subjected to the influences that are fitted to develop it, and, if the idea involved in this digression be correct, so will the talent, the arder, the industry, the skill, the knowledge of our profession, blossom forth when once it escapes from the darkness and blight of its long vassalage to antiquated forms, and obeys the stimulus of a higher science, and has the consciousness of its elevation and of its renewed power to fulfil the noble and beneficent purposes of its existence.

At the conclusion of the address a vote of thanks was accorded to the President.

MORE INVITATIONS.

An invitation was received from the Physicians of the French Hospital to visit that institution.

The members were invited to bring their ladies to the morning sessions of the Association.

An invitation was also received from the California Academy of Sciences to visit their rooms.

Out of a list of seventeen Special Committees, only the following made reports, which were referred to the Sections:—

On Criminal Abortion. By Dr. D. A. O'Donnell, of Baltimore. Referred to the Section on Practical Medicine and Obstetrics.

On Climatology and Epidemics of California. By Dr. F. W. Hatch, of Sacramento. Referred to the Section on Climatology and Epidemics.

On the Climate and Diseases of Mississippi. By Dr. J. P. Moore, of Yaroo City. Same reference.

On the Climate and Diseases of Minnesota. By Dr. Charles N. Hewitt, of Minneopolis. Same reference.

Several volunteer communications were received, and referred to the Sections on Surgery, and on Practical Medicine and Obstetrics.

One valuable paper, on the Medical Botany of California, was presented by Dr. Gibbons, of Stockton, Cal., and referred to the Section on Materia Medica and Chemistry.

On motion, the Association adjourned until ten o'clock on Wednesday morning.

---

SECOND DAY.

At half-past ten o'clock the Association met at Pacific Hall, Dr. Stillé, President, in the chair. The minutes of the first day's session were read and approved.

LIST OF DELEGATES.

The Committee of Arrangements presented a report of duly accredited delegates and permanent members.

Dr. A. E. Ames moved that the report be received, except that portion referring to the members by invitation.

Dr. Toner raised the point as to Dr. Thomas' relations to the Medical Society of Philadelphia.

Dr. Gibbons, Sr., suggested that the point raised might give rise to angry discussion, and he moved that the Chair appoint a Committee on Ethics, to whom such matters should be referred.

On being put to the vote, the motion was carried by a vote of 87 ayes to 15 noes.

The report of the Committee was on motion then received.

CALIFORNIA PHYSICIANS.

Dr. T. M. Logan submitted a list of members of the California State Medical Society not delegates, and moved that they be elected members by invitation.

The Chairman read the section of the law of the Association, which provides that members by invitation can only be elected when they are from districts unrepresented by delegates in the Association.

Dr. Stout said that notwithstanding the statute, he hoped some indulgence would be extended to the physicians of California.

Dr. Davis, of Chicago, strongly urged that the laws be not violated; they had been carefully prepared to cover that very point. He moved that the members of the California State Medical Society be invited to seats on the floor of the Convention, and mingle with the members of this Association.

Dr. Logan then withdrew his motion, and the one proposed by Dr. Davis was put to vote and carried.

MEDICAL EDUCATION.

At the request of the Secretary, Dr. Yandell came on the stage to read the report of the Committee on Medical Education, signed by Dr. E. Geddings, of South Carolina.

Dr. Toner objected to the report, on the ground that it was signed only by one member of the Committee.

The Chair decided the point not well taken.

An appeal was taken, and the decision of the Chair was sustained.

Dr. Yandell proceeded to read the report, which is a pamphlet of thirty-nine printed pages, but before he had concluded its reading, Dr. Gibbons moved that the further reading be dispensed with, and referred to the Committee on Publication, which motion, after a lengthy discussion, prevailed.

INVITATIONS.

Dr. Gibbons then announced the various invitations tendered to the Association, which have already been published.

A MEMBER REBUKED.

Dr. Gibbons, Sr., moved that the vote be reconsidered whereby the Committee on Vaccination was continued for another year, and that its Chairman, Dr. Henry Martin, be removed, for the reason that he had written a communication to a homœopathic journal in Massachusetts, attaching to it his official signature.

Dr. Storer suggested that the matter be referred to the Committee on Ethics.

Dr. Dawson said that the article was an insult to every member of the Association, and moved that Dr. Martin be expelled as a member of the Association.

Dr. Bibb offered an amendment, that a committee of three be appointed to prefer charges against the gentleman. It was finally resolved to refer the matter to the

COMMITTEE ON ETHICS, which was then appointed by the Chair, consisting of Drs. Gibbons, Davis, Smith, Toner, and Parsons.

On motion of Dr. Stout, it was resolved to refer all questions implying accusations to the Committee on Ethics without discussion.

PRIZE ESSAYS.

Dr. Logan presented a report from the Committee on Prize Essays, who state that they had received five essays, and that they award the prize to E. R. Taylor, of Sacramento City, for his essay on the "Chemical Constitution of the Bile," bearing the motto, "Divide et impera."

The second prize was awarded to B. M. Howard, of New York (winner of the first prize last year), for his essay on "The direct method of artificial respiration for the treatment of persons apparently dead from suffocation by drowning, or from other causes." Motto, "Festina lanta."

The report was adopted, and the Committee requested to hold all essays at the disposition of the authors.

RESOLUTIONS ADOPTED.

Dr. Davis then presented a lengthy report from the Committee on Correspondence with State Medical Societies, and submitted the following resolutions, which were adopted:

*Resolved*, That each State and Local Medical Society be requested to provide, as a permanent part of its organization, a Board of Census for determining the educational qualifications of such young men as propose to commence the study of medicine, and that no member of such societies be permitted to receive a student into his office until such student presents a certificate of proper preliminary education from the Census Committee appointed for that purpose, or a degree from some literary college of known good standing.

*Resolved*, That a more complete organization of the profession in each State is greatly needed for the purpose of affording a more efficient basis, both for educational and scientific purposes.

*Resolved*, That a Committee of three be appointed for the purpose of continuing the correspondence with the State Medical Societies, and of asking their earnest attention to the foregoing resolutions, in addition to those submitted for their action in 1869.

Dr. Moore, of St. Louis, offered a resolution that all Medical Colleges charge $100 as the fee for a course of lectures, and that a forfeiture of this rule shall subject such college to no representation in the Convention. After a protracted discussion, the resolution was voted down, on the ground that quality of education does not depend on price.

A recess of ten minutes was taken, to enable members from the different States to agree on a member to serve on the Committee on Nominations.

The Convention then adjourned.

---

THIRD DAY.

The third session of this National Association was held at Pacific Hall. Vice-President Gibbons, in the absence of the President, called the meeting to order.

THE VISIT TO OAKLAND.

Professor Carr, of Oakland, was introduced, and stated that the invitation to visit Oakland was extended by the Mayor, profession, and citizens of that city. The party would be taken from the Oakland wharf over the bay, where conveyances would be in waiting for them to show them around Oakland, and to partake of a collation of Californian products.

On motion of Dr. E. A. Ames, the invitation was accepted, and the Association decided to meet next day at 9 A.M., and adjourn at 11 A.M., to take the 11.10 boat for Oakland.

An expression being desired as to the number that would attend the trip, a vote was taken, and all present voted in the affirmative, and 25 per cent to be added for the ladies. The party will consist of about 200.

ADDITIONAL DELEGATES.

Dr. Stout stated that through inadvertence the following names had been omitted from the roll of delegates: Dr. Ariel B. Hovey, of Ohio; Alexander Fisher, of Chicago; G. George Tyrell, of Sacramento, and Dr. John Fiske, of Amador.

REPORTS.

A report was read from the Committee on Publication, giving an account of the labors performed by them last year.

The Treasurer presented his annual report, showing the receipts to have been $3,802 88; disbursements, $3,098 56; balance on hand, $704 32.

The annual report of the Librarian was next read, stating that the books of the Association were being well preserved at the Smithsonian Institute. At the last year's report the Library numbered 329 volumes, to which had been added during the year medical journals. The grand idea of the Association is to establish and form an American Medical Repository, where the whole medical literature of this country and continent might be preserved and owned by the representative Association of the profession of the new world.

The idea has as yet failed to be realized, but many are now manifesting an interest in it, and an appeal is made to American authors to furnish copies of their works to this Association. The reports were referred to the Committee on Publication.

INSANE ASYLUMS.

Dr. Kirwan, of Pennsylvania, presented a report from the Association of Superintendents of Insane Asylums, which was organized in 1844, which was to the effect that the Association could accomplish more good by having a distinct Association, at which all matters relative to the care and treatment of the insane could be fully discussed better than if referred to a section of this Association.

Dr. Storer offered the following resolution, which was adopted:

*Resolved*, That the Association of Superintendents of Institutions for the Treatment of the Insane and the American Medical Association should be more closely united, and that the meetings of the two Associations should be held about the same time and at the same place.

Dr. Y. C. Atlee, delegate from this Association to the Association of Superintendents of Insane Asylums, presented a report of his labors.

NATIONAL MEDICAL SCHOOL.

Dr. Johnson, of Missouri, presented a report from a Special Committee, suggesting a plan for the elevation of medical attainments and establishment of a National Academy of Medicine. Referred to Committee on Education.

NAVAL EDUCATION.

Dr. Yandell presented a report from the Special Committee, to examine Dr. Pinckney's report on the British system of naval medical education. Referred to Committee on Education.

SURGERY.

Dr. E. T. Barber, of Yreka, submitted a paper on the fracture of the neck of *fermur*, in a child seven years old. Referred to Committee on Surgery.

BOTANY OF CALIFORNIA.

Dr. Yandell, Chairman of the section on *Materia Medica* and Chemistry, reported that the only paper they had received was from Dr. W. P. Gibbons, of Alameda, on the Medical Botany of California, together with 180 specimens of indigenous. They considered it one of the most important ever presented to the Association, and moved its reference to the Committee on Publication. Dr. Gibbons moved to refer it back to the author, to be completed and presented next year. Carried.

THE CANADIAN ASSOCIATION.

Dr. Storer, delegate to the Canadian Medical Association, reported that he attended the sessions of that body at Ottawa, and was well received. He stated that most of the members were graduates of British Colleges, and their education was far above the average of the profession in this country. They extended an invitation to the Association to send delegates to its meetings.

The Committee on Ethics presented a report, which was received and accepted. They made no report in the case of Dr. Thomas, representative from a female college in Philadelphia, as the matter would come up under a proposed amendment to the Constitution.

FEMALE COLLEGES.

Under the head of unfinished business the following amendment to the Constitution, proposed at the last annual session by Dr. Henry Harthshorne, of Pennsylvania, was called up:

"Nothing in this Constitution shall be so construed as to prevent delegates from colleges in which women are taught and graduated in medicine, and hospitals in which medical women, graduates in medicine attend, from being received as members of this Association."

This drew forth an animated discussion *pro* and *con*, and a motion to indefinitely postpone the subject matter prevailed, by a vote of 80 ayes to 25 noes.

ELECTION OF OFFICERS.

Pending the discussion on the Woman question, the Committee on Nominations came and presented the following names as officers for the ensuing year: President, D. W. Yandell, of Kentucky; First Vice-President, T. M. Logan, of California; Second Vice-President, C. L. Ives, of Connecticut; Third Vice-President, R. M. Michel, of Alabama; Fourth Vice-President, J. K. Bartlett, of Wisconsin; Permanent Secretary, W. B. Atkinson, of Pennsylvania, who is elected for life; Assistant-Secretary, D. Murray Cheston, of Pennsylvania; Librarian, F. A. Ashford, District of Columbia; Treasurer, Casper Wistar, of Philadelphia.

On motion of Dr. Davis, the report was received, and the gentlemen named unanimously elected.

The Convention then adjourned to Toland Medical College, being conveyed thither in cars.

The guests were cordially received at the College by the Faculty. They were then shown through the institution, which was highly complimented as being one of the most complete and best arranged in the country. A sumptuous collation was spread and partaken of with gusto. Toasts were proposed and responded to, and after spending two hours agreeably, the guests returned to their hotels well pleased.

---

FOURTH DAY.

The Association met, pursuant to adjournment, at 9 A.M., President Stillé in the Chair. The minutes of the previous meeting were read and approved.

THANKS FROM CALIFORNIA.

The following was read, and ordered spread on the minutes:

OFFICE OF CALIFORNIA STATE MEDICAL SOCIETY,
SAN FRANCISCO, *May 5th, 1871.*

*To the Members of the American Medical Association:*

GENTLEMEN,—Allow me, in the name of the Medical Men of the Pacific Coast, to tender our thanks to the Association for the honor conferred upon us by the assembling of members of this Association in the city of San Francisco, whereby so many of our brethren, who for years have been isolated from the Association of their more favored Eastern brethren, have had an opportunity of attending the deliberations of this honored and national body.

GEORGE HUESTON,
*Corresponding Sec. of the State Medical Asso.*

CHAIRS OF HYGIENE TO BE ESTABLISHED.

Dr. T. M. Logan, of Sacramento, offered the following resolutions, which were adopted:

*Whereas,* The science of Hygiene and its corollary preventive, a State medicine, are subjects eminently congenial with the purposes of this Association, inasmuch as they have for their objects the preservation of human life, and the removal of those causes of disease and death which it is in the power of legislation to ameliorate, if not eradicate; and, whereas, the great fundamental idea that was made the prominent element for medical association, and that led eventually to our national organization, was a higher standard of medical education; and, whereas, the present system adopted by our Colleges provides more and more satisfactorily for the thorough qualification of the graduate, as regards the principles and practice of his art, but does not provide at all adequately for the special study and cultivation of questions of State medicine; therefore be it

*Resolved,* That this Association recommends a distinct and separate chair of hygiene, independent of physiology, to be established in all our medical schools, and constituted a requisite curriculum preliminary to that diploma which confers one of the highest honors of the profession.

*Resolved*, That the inauguration of the enlarged philanthropic policy of State medicine in Massachusetts and California is worthy of our special approbation, and commends itself to other States for imitation; and, therefore, the President of this Association is hereby authorized to nominate at this session, a Committee, consisting of one physician from each State in the Union, to memorialize the Legislatures of all the other States to follow the example of one of the oldest, most enlightened, and conservative, as well as one of the youngest, most progressive, and enterprising members of our glorious confederacy, who have led off in the right way, and at the right time, for the prevention of disease and the correction of "those multitudinous agencies, whether physical, whether moral, whether born of earth, of air, or of society, which are either openly or insidiously degenerating the human race."

*Resolved*, That this Association further recommends that initiative steps be taken, as soon as six States shall engraft State Medicine upon their statute books, for the formation of a "National Health Council," whose objects shall be the prosecution of the comparative study of international hygienic statistics, and the diffusion and utilizing of sanitary knowledge; and that said Counil shall be aided and assisted by this Association in using whatever influence may legitimately lay in their power, with foreign States, as well as with the medical profession, and the people generally, in securing co-operation in the ends and objects of public hygiene.

*Resolved*, That said National Health Council, although thus organized as a branch *per se*, shall be auxiliary to this Association, and shall constitute a special section to hygiene, to which all questions, germane to this department of medicine, shall be referred. "Only," to use the language of the great Virchow, "by thus working harmoniously together, by thus mutually enlightening each other, will the State gain an organ to which may be safely intrusted the solution of the great question of our time, viz.: bodily and mental health, and development of future generations.

### OFFICERS FOR THE SECTIONS.

Chemistry and *Materia Medica*—Prof. R. E. Rogers, Philadelphia, President; E. Cutter, Mass., Secretary.

Practical Medicine and Obstetrics—D. A. O'Donnel, Baltimore, President; B. F. Dawson, N.Y., Secretary.

Surgery—John T. Hodgen, Missouri, President: W. F. Peck, Davenport, Iowa, Secretary.

Meteorology and Epidemic Diseases—George Sutton, Ind., President; Elisha Harris, N.Y., Secretary.

Medical Jurisprudence—S. C. Busey, Washington, President; E. L. Howard, Baltimore, Secretary.

Physiology—J. C. Dalton, N.Y., President; D. Payton, Oregon, Secretary.

Psychology—Isaac Ray, Philadelphia, President; John W. Kirwin, Pennsylvania, Secretary.

Library Committee at Washington—Dr. J. M. Toner.

COMMITTEES FOR THE ENSUING YEAR.

The Committee on Nominations presented a report of Committees for the ensuing year, which, on motion, was adopted:

Committee on Publication—Dr. F. G. Smith, of Pa., Chairman; W. B. Atkinson, Pa.; D. Murray Cheston, Pa.; F. A. Ashford, D.C.; Caspar Wistar, Pa.; H. F. Askew, Delaware; I. Aitken Meigs, Pa.

Committee on Prize Essays—Dr. A. Stillé, Chairman, Phila.; F. G. Smith, Phila.; D. A. O'Donnell, Balt.; B. F. Dawson, N.Y.; L. P. Bush, Dela.

Committee on Medical Education—J. S. Wetherly, Alabama, Chairman; L. Cooper Lane, S.F.; J. M. Toner, Washington; Samuel Wiley, Minn.; W. O. Baldwin, Alabama.

Committee on Medical Literature—T. Parvin, Ind., Chairman; ——— Carpenter, Oregon; J. P. Whitney, S. F.; G. Mendenhall, Cincinnati; L. P. Garvin, R.I.

Committee on American Medical Necrology—Chairman, John D. Jackson, Ky.; Chas. W. Parsons, R.I.; E. A. Hildreth, West Va.; Wm. Lee, Washington, D.C.; T. M. Logan, Cal.; W. C. Warrender, Oregon; H. D. Holton, Vt.; W. J. Scott, Ohio; W. D. Buck, N.H.; A. Sagar, Michigan; V. Karsey, Ind.; A. E. Ames, Minn.; H. K. Steele, Col.; ——— Mason, Wis.; S. D. Gross, Phila.; D. W. Stormont, Kansas; J. B. Johnson, Mo·; H. R. Storer, Mass.; H. W. Rushenburger, U.S. Navy; I. W. H. Baker, Iowa; J. O. Hamilton, Ill.; ——— Peabody, Neb.; L. P. Bush, Del.; G. W. Russell, Conn.; Paul C. Chew, Md.

Committee of Arrangements—Dr. E. Hartshorne, Chairman; Drs. S. W. Gross, Murray Cheston, J. F. Maury, James Tyson, S. W. Mitchell, John H. Packard, William Pepper, Richard Townsend.

On the Climatology and Epidemics of—Maine, Dr. Wood, Portland; New Hampshire, A. B. Crosby; Massachusetts, E. Cutter; Rhode Island, Edward T. Caswell; Connecticut, I. C. Jackson; New York, W. F. Thomas; New Jersey, E. M. Hunt;

Pennsylvania, W. S. Wells; Maryland, C. H. Ohr; Georgia, A. I. Senimes; Missouri, W. S. Edgar; Alabama, R. T. Mitchell; Texas, S. M. Welch; Illinois, D. Prince; Indiana, D. Clark; District of Columbia, J. W. H. Lovejoy; Iowa, I. Williamson; Michigan, Douglas; Ohio, J. A. Murphy; California, F. W. Hatch; Tennessee, B. K. Bowling; W. Virginia, E. A. Hildreth; Minnesota, Charles N. Hewitt; Virginia, Wortham; Delaware, L. P. Bush; Arkansas, Sinks; Mississippi, J. P. Moore; Louisiana, S. M. Bemiss; Wisconsin, J. K. Bartlett Kentucky, L. P. Yandell, Sr.; Oregon, E. R. Fisk; North Carolina, F. J. Haywood; Colorado, R. G. Buckingham; South Carolina, M. Simmons.

Special Committees—Dr. A. L. McArthur, Chicago, Illinois. On the nature and process of the restoration of Bone.

George Sutton, Indiana. Comparative Pathology and the effects which diseases of inferior animals have upon the human system.

Dr. Antisell, Chairman of the Committee on the cultivation of the Cinchona Tree.

Vaccination—Chairman, Dr. T. M. Wise, Kentucky.

Anatomy and Disease of the Retina—R. F. Michell, Alabama.

Some Diseases peculiar to Colorado—John Elsner, Denver, Colorado.

Skin Transplantation—J. Ford Thompson, Washington, D.C.

SECTIONS.

The Secretary presented the report of the Section on Practice of Medicine and Obstetrics, submitting papers on Criminal Abortion, by Dr. D. A. O'Donnell, of Maryland, and on the Social Evil, by Dr. Stout, of San Francisco. Referred to the Committee on Publication.

Dr. O'Donnell read a series of resolutions condemning in the strongest terms abortionists, and resolving to hold no intercourse with them. The resolutions were adopted.

THE NAVAL CORPS.

Surgeon J. M. Browne, U.S.N., on behalf of the medical corps of the Navy, returned the gratitude of the corps to the Association for the aid extended to them in the contest between the line and staff, which, by a recent Act of Congress, defines their position and rights, with equality of rank and to the dignity of the profession. Though it was not all they had desired, yet still it was a fair compromise, and the situation ought to be accepted by all parties. He then read some resolutions adopted by the representatives of the Navy, expressing their thanks for the sympathy extended to them in the protracted struggle, and inviting young men to enter the Navy. The resolutions were referred to the Committee on Publication.

ETHICS.

Dr. Montgomery offered a resolution that a Chair of Ethics be established in all Medical Schools of the country. It was subsequently withdrawn.

PHYSICIANS IN THE UNITED STATES.

Dr. M'Arthur moved that Dr. Toner be requested to furnish to the Association an abstract of the statistics prepared by him with care and labor. It was a list embracing the names of 60,000 physicians in this country, the number that paid revenue licenses, and 3000 of which were homeopaths. Referred to the Committee on Publication.

MEMORIAL FUND.

Dr. Storer moved that the Association take some action to raise funds towards the Memorial Fund inaugurated by the physicians of Europe in honor of the late Sir James Y. Simpson, of Edinburgh. Adopted.

REPRESENTATIVES OF FEMALE COLLEGES.

Dr. Atlee, of Philadelphia, offered the following resolution: *Resolved*, That the American Medical Association acknowledges the right of its members to meet in consultation the graduates and Teachers of Women's Medical Colleges, provided the code of ethics of the Association is observed.

An animated discussion ensued on this proposition, but the hour for adjournment to Oakland (11 A.M.) having arrived, the Convention, on motion, adjourned until evening.

EVENING SESSION.

The Association reassembed at 8 P.M.

In the absence of the Secretary, Dr. J. C. Tucker acted as Secretary *pro tem.*

The Chairman stated the first business in order, was the resolution offered by Dr. Atlee prior to the adjournment of the morning session, and then read the resolution.

This was followed by a very hot debate; and the matter finally disposed of by being indefinitely postponed.

THANKS RETURNED.

Resolutions were offered and adopted returning thanks to the various railroad lines, and to all individuals and associations who had extended courtesies to the members of the Association; also, to the President and Secretary for the faithful discharge of their duties.

MORE INVITATIONS.

Invitations extended by Dr. Shurtliff, Superintendent of the Insane Asylum at Stockton, for the Association to visit that institution; and also from the Mercantile Library Association, extending the freedom of the rooms, etc., to members during their stay in this city.

ADJOURNMENT.

Dr. Davis, in a few appropriate remarks, spoke of the pleasant reunion they had during the sessions, the good feeling existing, of the hospitalities they had received, and then moved to adjourn *sine die*, which was, on being put to vote, carried.

